# Patterns of Pronoun Affirmation Across Social Contexts and Opportunities for Resilience Among Trans and Nonbinary Youth

**DOI:** 10.3390/bs16101756

**Published:** 2026-09-27

**Authors:** Alyssa N. Garcia, Russell B. Toomey, Roberto L. Abreu

**Affiliations:** 1 Norton School of Human Ecology, Human Development and Family Science, The University of Arizona, Tucson, AZ 85721, USA; 2 Department of Psychology, University of Florida, Gainesville, FL 32611, USA

**Keywords:** trans and nonbinary youth, pronoun affirmation, gender affirmation, resilience, positive youth development

## Abstract

This study examined chosen pronoun affirmation across multiple social contexts (i.e., home, online school, in-person school, work, friends, and extended family) as a predictor of mental health among 319 trans and nonbinary (TNB) youth (ages: 13–22; *M_age_* = 18.6, SD = 2.18). Pronoun affirmation is increasingly restricted, particularly in schools, as part of widespread anti-trans legislation in the U.S. Guided by an integration of resilience, minority stress, and intersectionality frameworks, this study used a person-centered approach (i.e., latent class analysis), identifying three distinct affirmation classes: predominantly friends affirmation (27.3%), non-familial affirmation (55.8%), and comprehensive affirmation (16.9%). Transmasculine youth were more likely to be represented in comprehensive affirmation, while nonbinary youth were less likely to be represented in the comprehensive affirmation class. Compared to youth in the predominantly friends affirmation class, youth in non-familial affirmation and comprehensive affirmation classes reported significantly lower depressive and anxiety symptoms, better future outlook, and lower odds of suicide thoughts. In variable-centered analyses, home affirmation was associated with fewer depressive and anxiety symptoms, online and in-person school affirmation was associated with all outcomes except suicide attempts, friend affirmation was similarly associated with all outcomes except suicide thoughts and attempts, and extended family affirmation was associated with lower depressive and anxiety symptoms and lower odds of suicide thoughts. Findings underscore the importance of pronoun affirmation across institutional, peer, and family contexts and carry direct implications for school policy, family-based interventions, and advocacy amid increasing legislative restrictions on gender-affirming practices.

## 1. Introduction

Trans and nonbinary (TNB) youth encounter several challenging conditions that seek to limit their full potential and resilience (Brooks, 1981; Meyer, 2003, 2007; Toomey, 2021). Due to these structural and interpersonal challenges, TNB youth report significant disparities across multiple outcomes compared to their cisgender peers, including heightened depressive and anxiety symptoms, and experiences of suicide thoughts and attempts (Gordon et al., 2024). Studies have indicated, however, that resilience and thriving are possible for TNB youth, particularly when gender affirming actions are taken in a variety of social contexts (Russell et al., 2018; The Trevor Project, 2022, 2024). That is, individuals in youths’ immediate contexts (e.g., family, peers) may engage in gender affirming actions and behaviors that convey acceptance (or rejection when affirming behaviors are not present) of TNB youths’ authentic selves (i.e., gender identity; Ehrensaft, 2017; Meyer, 2003, 2007), which are related to resilience and well-being. One critical component of gender identity affirmation involves others’ consistent and intentional use of TNB youths’ chosen pronouns (e.g., he/him, she/her, they/them, or other pronouns that reflect an individual’s gender identity rather than pronouns assigned at birth; Perry & Pauletti, 2011). Although few studies have examined associations between affirmation of chosen pronouns and well-being among TNB youth, existing studies have demonstrated that use of chosen names and pronouns can serve as a promotive factor for mental health and well-being (Russell et al., 2018; The Trevor Project, 2022, 2024). Conversely, when chosen pronouns and names are not used across contexts, TNB youth may experience heightened psychological distress and engage in harmful coping behaviors (e.g., suicide thoughts and attempts; McLemore, 2018; Price & Green, 2023). Understanding the specific role that pronoun affirmation plays in these outcomes is crucial, especially given the current sociopolitical climate where anti-LGBTQ+ legislation (with a particular focus on anti-trans policies; Movement Advancement Project, 2025) is increasingly restrictive of gender-affirming practices in schools and other contexts (Goldberg et al., 2024).

While existing research has established associations between gender affirmation and mental health outcomes, there remains a need to specifically examine how pronoun affirmation across multiple socioecological contexts relates to better mental health outcomes (such as endorsing a positive future outlook) and poor mental health outcomes (depressive and anxiety symptoms and suicide thoughts and attempts among TNB youth). Further, the current sociopolitical climate has led to restrictive legislation and several state laws targeting inclusive pronoun use within classrooms and schools (Glisten, 2026; GLSEN, 2023; Hoover & Bostic, 2021). Most existing research relies on variable-centered approaches that assume homogeneity among TNB youth and may obscure our ability to identify distinct profiles of pronoun affirmation across socioecological contexts that differentially contribute to mental health outcomes (Laursen & Hoff, 2006; Russell & Fish, 2016; Sequeira et al., 2022; The Trevor Project, 2022). The current study addresses this gap by examining (a) patterns of pronoun affirmation across socioecological contexts using a person-centered approach (i.e., latent class analysis), and (b) the unique contributions of pronoun affirmation within socioecological contexts using person-centered and variable-centered approaches, and their associations with mental health outcomes among TNB youth.

### 1.1. Pronoun Affirmation and Mental Health

Among TNB youth, the adolescent period involves navigating identity development within heterogeneous social contexts that include those that are affirming and non-affirming (Conley et al., 2023; Meyer, 2003, 2007). Pronoun affirmation refers to the consistent and respectful use of an individual’s chosen pronouns by individuals in their social contexts. Research demonstrates that other forms of gender affirmation (e.g., chosen name use) are associated with better mental health outcomes for TNB youth. Russell et al. (2018) found that TNB youth who could use their chosen names across multiple contexts (home, school, work) experienced significantly lower levels of depression and suicide thoughts compared to those with more limited contexts of affirmation. Similarly, Sequeira et al. (2022) found that TNB youth who reported higher levels of pronoun affirmation across multiple contexts reported significantly better mental health outcomes compared to those experiencing less affirmation. Together, these findings highlight the cumulative protective effect of affirmation across multiple social contexts.

Alternatively, when TNB youth experience consistent misgendering, meaning individuals in a social context do not use youths’ chosen names and pronouns, they may internalize these negative messages about their identity (Russell & Fish, 2016) and experience heightened psychological distress (McLemore, 2018). Bauer et al. (2015) also found that pronoun rejection contributed to internalized distress, thus contributing to heightened depressive symptoms and experiences of suicide thoughts among TNB individuals. Further, McLemore (2018) assessed psychological effects of chosen pronoun rejection among TNB individuals, finding that repeated rejection was associated with greater risk for engaging in suicide attempts as a form of coping (Price & Green, 2023).

### 1.2. Pronoun Affirmation Across Social Contexts

The extensiveness of social contexts in which pronoun affirmation occurs likely contributes to TNB youths’ mental health outcomes. While research suggests that experiencing affirmation in any one context is positive, it is the range of affirming contexts that may be particularly protective for TNB youths’ well-being. Thus, pronoun affirmation across multiple contexts is likely to shape the well-being of TNB youth in meaningful ways compared to the experience of only being affirmed in any one particular context (e.g., school versus home).

Family contexts play a particularly crucial role in shaping mental health outcomes among TNB youth (Abreu et al., 2024; Brown et al., 2020). More specifically, Simons et al. (2013) assessed relationships between parental support and mental health outcomes for TNB youth, finding that greater pronoun affirmation by parents was associated with lower levels of depression and suicide attempts. This research underscores the importance of family acceptance in promoting healthy identity development and reducing psychological distress. Further, Olson et al. (2016) examined mental health outcomes among youth who received gender-affirming support from families compared to those who did not. Their research found that socially affirmed youth reported depression and anxiety symptoms comparable to their cisgender peers, suggesting that family affirmation serves as a powerful protective factor that reduces disparities in negative mental health outcomes for TNB youth. Moreover, family affirmation for sexual and gender minority youth is predictive of heightened self-esteem and lower risk for suicide attempts (Ryan et al., 2010).

Within school settings, chosen name use by teachers and school staff has been associated with fewer depressive symptoms and suicide thoughts (Russell et al., 2018). Chosen pronoun use by school staff has also been found to promote a sense of school belonging (Glisten, 2026; GLSEN, 2023). Russell and Fish (2016) noted that schools that implement gender-affirming policies, including training for educators on chosen pronoun use, contributed to safer and more accepting learning environments for TNB youth. Peer affirmation more broadly (e.g., supportive peer environments) has been linked to healthy self-esteem and better mental health outcomes (McGuire et al., 2010), though research specifically examining peer pronoun use remains limited. Together, these findings highlight the importance of pronoun affirmation across multiple contexts toward promoting positive mental health outcomes and preventing suicide risk for TNB youth. This study extends this work by examining chosen pronoun affirmation specifically, rather than gender affirmation broadly, across six socioecological contexts, using both person-centered and variable-centered approaches to identify how patterns of affirmation across family, peer, and school contexts uniquely contribute to mental health among TNB youth.

### 1.3. Resilience, Minority Stress, and Intersectionality

Resilience frameworks suggest that positive youth development occurs through dynamic interactions among youth and their social contexts (Lerner et al., 2023; Masten, 2014), supported by promotive resources in youths’ family and peer contexts (e.g., schools, communities; Zimmerman et al., 2013). In these models, individuals who experience supportive assets (externally and internally) are theorized as likely to thrive given the presence or abundance of supportive factors in their ecological contexts. We conceptualize pronoun affirmation as a promotive resource; a condition of the socioecological context that supports positive adaptation rather than as resilience itself. The mental health outcomes examined (depressive symptoms, anxiety symptoms, future outlook, suicide thoughts, suicide attempts) represent indicators of adjustment that may reflect resilience processes, but we acknowledge that lower symptom scores are not comprehensive indicators of TNB youth resilience.

For TNB youth, pronoun affirmation may function as a promotive factor through ways of expression recognition, acceptance, respect, and belonging across developmentally salient social contexts. Indeed, Hall et al. (2024) describe how having one’s gender correctly perceived and referred to by the public can contribute to gender congruence, which ultimately can lead to gender euphoria, or the joy of engaging with one’s internally felt gender. Toomey (2021) further hypothesized that interpersonal gender dysphoria, or the lack of affirmation from others related to one’s gender, can contribute in meaningful ways toward TNB youth development and well-being. Thus, pronoun affirmation among TNB youth is likely a key aspect of one’s gendered experiences that contribute to resilience, even in the context of other minority stressors related to gender.

Minority stress theory (Brooks, 1981; Meyer, 2003, 2007; Toomey, 2021) can help explain the unique positions, identities, and experiences that TNB youth navigate, and how pronoun affirmation across contexts is associated with resilience among TNB youth. Minority stress theory suggests that stressors experienced in response to both systemic (e.g., anti-trans legislation) and interpersonal (e.g., lack of pronoun affirmation in school) factors are chronic and can compound and contribute to negative outcomes.

Given the historical and persistent injustices that TNB populations have experienced (Santos & Toomey, 2018), it is important to incorporate Crenshaw’s intersectionality framework (Crenshaw, 1991) as an interpretive lens to situate our findings within the context of multiple marginalized identities. Rather than engaging intersectionality as a fully operationalized analytic strategy, which would require intersectional subgroup analyses or interaction terms beyond the scope of the current study, we use intersectionality to inform our interpretation of how identity and social location may shape differential access to pronoun affirmation across social contexts. An intersectional lens can provide the structure to examine and explore how oppression is not experienced homogeneously for all members of a population, given that individuals hold multiple overlapping oppressed and privileged identities across social locations. For example, TNB youth are not only navigating cissexist systems of oppression of gender identity, but also heterosexist, racist, and ableist systems, among others. Additionally, as minors, they have less bodily autonomy and agency compared to adults (Wray-Lake et al., 2025), which has been made more salient by recent bans on inclusive language in classrooms (Movement Advancement Project, 2025).

Given that contexts are heterogeneous in terms of affirmation of TNB identities and expressions, TNB youths’ chosen pronouns are potentially respected in some contexts but not others. For example, TNB youth might experience affirmation in inclusive contexts (e.g., LGBTQ+ school clubs), and experience harmful rejection of chosen pronouns in other contexts (e.g., anti-trans ideology expressed in certain contexts of faith or culture). Relatedly, TNB youth, who are more likely to have a disability than cisgender youth (The Trevor Project, 2023), might experience support for their disability in some spaces, while simultaneously experiencing rejection of their chosen pronouns within that same space. As an example, the U.S. Trans Survey (2022) estimated that 58% of TNB youth experienced negative health care experiences related to their disabilities (Rastogi et al., 2025). These intersectional experiences can contribute to more stress and greater vulnerability to poor mental health outcomes (Santos & Toomey, 2018). By applying resilience, minority stress, and intersectionality frameworks, we aim to understand how affirming pronoun use can serve as a promotive factor for resilience in TNB youth and examine heterogeneity in these associations by additional oppressed identities and experiences.

### 1.4. Current Study

In response to increasing anti-LGBTQ+, especially anti-trans-focused, legislation and growing concerns about sexual and gender minority youth well-being (Goldberg et al., 2024), there is an urgent need to understand how pronoun affirmation across multiple social contexts relates to resilience among TNB youth. While existing research has established the importance of gender affirmation for TNB youth and well-being, several gaps remain. First, much of the existing research focuses on general gender affirmation (e.g., chosen name use, Russell et al., 2018) rather than specifically examining chosen pronoun use across social contexts. Second, there is limited research examining patterns of pronoun affirmation across contexts and how these patterns relate to mental health outcomes. Most existing research uses variable-centered approaches that treat TNB youth as a homogeneous group, potentially obscuring distinct patterns of pronoun affirmation across contexts that may differentially contribute to mental health outcomes (Russell & Fish, 2016; Sequeira et al., 2022; The Trevor Project, 2022). The current study examines how pronoun affirmation differs across contexts, how distinct classes of pronoun affirmation emerge, and how pronoun affirmation classes are associated with better mental health outcomes among TNB youth. Further, in addition to a person-centered approach, we also examine how individual pronoun affirmation contexts are uniquely associated with mental health among TNB youth in order to identify all possible policy and practice intervention points for future research. Specifically, the current study tested the following hypotheses guided by our research questions:

*RQ*_1_: How does pronoun affirmation differ across social contexts among TNB youth?

**H_1_.**  *We expected that pronoun affirmation would differ across contexts, with affirmation reported more frequently in peer and school contexts and less frequently in family contexts.*

*RQ*_2_: What are the latent classes of pronoun affirmation across contexts among TNB youth?

**H_2_.**  *We expected that distinct pronoun affirmation classes would emerge across contexts, reflecting different patterns of affirmation in family, peer, and school contexts, although given the exploratory nature of this type of analysis, we did not hypothesize the number or distinct types of classes that would emerge.*

*RQ*_3_: How do pronoun affirmation classes differ by gender identity, sexual orientation, race/ethnicity, age, and disability status?

**H_3_.**  *Framed by intersectionality perspectives (Santos & Toomey, 2018), we expected that youth with multiple oppressed identities and locations would be more represented in lower affirmation classes, and given our limited sample size, these associations were examined via individual demographic covariates rather than modeled intersections.*

*RQ*_4_: How do pronoun affirmation classes relate to mental health outcomes, including future outlook, depressive symptoms, anxiety symptoms, and suicide thoughts and attempts, among TNB youth?

**H_4_.**  *We expected that higher affirmation classes would be associated with better mental health outcomes, including more positive future outlook, lower depressive and anxiety symptoms, and fewer suicide thoughts and attempts.*

*RQ*_5_: Given limited prior research directly comparing specific contexts of pronoun affirmation among TNB youth, additional variable-centered analyses examined how individual contexts of pronoun affirmation were uniquely associated with mental health. These exploratory analyses were guided by the uniqueness of institutional and peer contexts, the salience of these environments during adolescence, and the disproportionate impact of recent anti-trans legislation targeting schools.

## 2. Methods

### 2.1. Procedure

Participants were recruited through Qualtrics Panel Services in Spring 2022 to complete a web-based survey aimed at assessing the relationship between family and peer support and mental health outcomes among TNB youth. Qualtrics Panel Services members were recruited from several sources, including social media, and members were validated by third-party verification. Members are monetarily compensated for their participation in the panels by Qualtrics, and a compensation contract is agreed upon between members and Qualtrics. Assent and consent were received prior to data collection; a waiver of parental consent was granted in order to protect youth who were not out to their parents. The study protocol was approved by the University of Arizona Institutional Review Board.

### 2.2. Participants

The current study included 319 TNB youth, ages 13–22 years (*M_age_* = 18.6, *SD* = 2.18). Regarding racial/ethnic identity, about 43% identified as Youth of Color—operationalized as a binary indicator, consistent with prior research examining racial minority stress among TNB youth (Bauer et al., 2021), including 17.6% Latinx; 14.7% Black; 9.1% multiracial/ethnic; 6.3% Asian; and 2.2% Indigenous/Native American—and 60.8% identified as White. Within our sample, 77.1% of participants identified as nonbinary; 23.2% identified as transmasculine; 10% identified as transfeminine; and 15.7% of participants identified as other gender identity. Participants were able to select all applicable gender identities, and percentages therefore sum to greater than 100%. Regarding sexual orientation, 19.4% of participants identified as monosexual (5.3% gay; 11.6% lesbian; 2.5% heterosexual) and 78% identified as plurisexual (26% bi; 26.3% pan; 3.8% omni; 21.9% queer). Disability status was reported by 39.8% of the sample.

### 2.3. Measures

**Pronoun Affirmation in Multiple Contexts.** Pronoun affirmation in multiple contexts was assessed by participants responding to a single prompt stem (“Are you able to go by your chosen pronouns in the following places?”) across six contexts where pronoun affirmation might occur: (1) “*at home*”, (2) “*at school [online/remote]*”, (3) “*at school [in*-*person]*”, (4) “*at work*”, (5) “*with friends*”, and (6) “*with extended family*”. Participants indicated whether individuals in each context had used their chosen pronouns: *No* (0), *Yes* (1), and *Not Applicable* (-99). Participants who indicated a context was not applicable were coded as missing for that indicator. In order to account for contexts that were reported as not applicable to some participants, a percentage score was created for descriptive analyses by dividing the total number of affirmed contexts by the total number of applicable contexts for each participant. For the latent class analysis, binary indicators (0 = *not affirmed*, 1 = *affirmed*) of pronoun affirmation were used to identify patterns of affirmation across contexts. Because the work context had a high degree of non-applicability (55%), five indicators were retained for analyses: home, online school, in-person school, friends, and extended family.

### 2.4. Mental Health

**Depressive and Anxiety Symptoms.** Depressive and anxiety symptoms were assessed using the National Institutes of Health’s Patient-Reported Outcomes Measurement Information System Scale–Short Form measures (Quinn et al., 2014). Eight items assessed depressive symptoms (e.g., “*I felt lonely*”), and eight items assessed anxiety symptoms (e.g., “*I got scared really easily*”). Response options ranged from *Never* (1) to *Always* (5). This measure has been validated in psychometric studies and demonstrated strong internal consistency in previous research (α = 0.95 for both subscales; Irwin et al., 2010). Internal consistency was maintained in the current sample (α = 0.95; ω = 0.95 for both depressive and anxiety symptoms).

**Future Outlook.** Participants’ future outlook was assessed using the Hopeful Future Expectations Scale (Bowers et al., 2014) with a single prompt stem: “*Think about how you see your future. What are your chances for the following?*” Four items assessed future outlook (e.g., “*having friends you can count on*”). Response options ranged from *Very Low* (1) to *Very High* (5). Internal consistency was acceptable (α = 0.69; ω = 0.70).

**Suicide Thoughts and Attempts.** Past-year suicide thoughts were assessed with a single item: “*During the past 12 months*, *have you seriously thought of taking your own life?*” Response options were *No* (0) and *Yes* (1). Past-year suicide attempts were assessed with a single item: “*During the past 12 months*, *have you attempted to take your own life?*” Response options were *No* (0) and *Yes* (1). These items were adapted from the Centers for Disease Control and Prevention Youth Risk Behavior Survey Questionnaire (CDC, 2021), a widely used national survey in which single-item suicide assessments are best practice given the respondent burden associated with more extensive survey batteries in youth samples, including TNB youth research (The Trevor Project, 2022, 2024). Both suicide thoughts and suicide attempts items demonstrated medium-to-large positive correlations with depressive symptoms, supporting their validity.

### 2.5. Analytic Strategy

All analyses were conducted in R (version 4.6.0; R Core Team, 2026) using the *poLCA* package (version 1.6; Linzer & Lewis, 2011) to identify latent classes of pronoun affirmation patterns across five binary context indicators: home, online school, in-person school, friends, and extended family. To minimize the risk of local maxima, 50 random starting sets were used for each model, and the solution with the highest log-likelihood was retained. A series of models was tested with an increasing number of possible classes. Each model was compared using fit indices (e.g., Akaike Information Criteria [AIC], Bayesian Information Criteria [BIC], entropy) and interpretation based on existing research and theory to determine the appropriate number of classes retained. Although the 4-class solution produced a slightly lower AIC, the 3-class solution was retained based on lower BIC, acceptable entropy (0.724), strong average posterior probabilities across classes (0.993, 0.970, 0.888), a larger minimum class size, and more interpretable profiles. Local independence was assessed by examining bivariate residuals, and no substantial violations were detected. Class membership was assigned using modal posterior probability assignment. Descriptive statistics were conducted to characterize the sample. Chi-squared tests were conducted to examine demographic differences across latent classes, including differences by gender identity, race and ethnicity, sexual orientation, age, and disability status. One-way ANOVAs with Tukey’s honest significance difference (HSD) post hoc comparisons examined class differences in depressive and anxiety symptoms, and future outlook. Chi-squared tests examined class differences in suicide thoughts and attempts. Linear and logistic regression models tested the associations of pronoun affirmation class membership and outcomes, controlling for covariates. Linear regression models examined depressive and anxiety symptoms, and future outlook. Logistic regression models examined suicide thoughts and attempts. Predominantly friends affirmation served as the reference group across models. As additional analyses, context-specific regression models examined associations between each individual context and outcome, with one affirmation context entered at a time alongside covariates. Study covariates included gender identity (transmasculine identity [1 = indicated, 0 = not indicated]; transfeminine identity [1 = indicated, 0 = not indicated]; nonbinary identity as reference), race and ethnicity (Youth of Color = 1; White as reference = 0), sexual orientation (Plurisexual = 1; Monosexual as reference = 0), age (continuous), and disability status (Disability = 1; No Disability as reference = 0). Gender identity was operationalized as three non-mutually exclusive binary indicators (transmasculine, transfeminine, and nonbinary), with nonbinary identity used as a comparison condition to compare youth who did not endorse transmasculine or transfeminine identity. All other covariates were coded as binary indicators, where 0 = *not indicated*, and 1 = *indicated.*

Missing data were minimal (<1%) given that Qualtrics Panel Services replaces surveys that have even moderate amounts of missing responses to survey items. Given minimal missingness, analyses were conducted using listwise deletion methods (Enders, 2010). For the latent class analysis, *poLCA* was run with na.rm = FALSE, which retains participants with missing data on one or more indicators and uses available data for parameter estimation, resulting in an analytic sample of 319 participants, with 218 participants having fully observed data across all five indicators. For variable-centered regression models, listwise deletion was used within each model. The simultaneous model (Model B) resulted in *n* = 213 due to listwise deletion across all five contexts. To aid interpretation, standardized coefficients (β) are reported first in the text for linear regression models, followed by unstandardized coefficients (*B*) and standard errors (*SE*). Full statistics including *B*, *SE*, *β*, and significance levels are reported in the Tables. Adjusted odds ratios (AORs) and 95% confidence intervals (CIs) are reported for logistic regression models. A sensitivity analysis was also conducted that included the work context pronoun affirmation indicator as a sixth indicator to examine whether exclusion of the work context affected the latent class solution. Results are presented in Supplementary Tables S1 and S2; exclusion of this variable did not result in substantive changes to class identification or meaning.

## 3. Results

### 3.1. RQ_1_: Descriptive Statistics

On average, TNB youth reported that they were able to use their chosen pronouns in 57.4% of applicable contexts (*SD* = 29.18%). Context-specific affirmation rates varied (see Table 1). Pronoun affirmation contexts were most frequently reported with friends (94.9%, *n* = 297). Pronoun affirmation was also common in school contexts (in-person: 66.8%, *n* = 177; online: 66.5%, *n* = 163). Pronoun affirmation was less frequently reported at home (41.3%; *n* = 128), and least frequently reported with extended family members (19.3%; *n* = 57). TNB youth reported moderate depressive symptoms (*M* = 3.13, *SD* = 1.14), anxiety symptoms (*M* = 2.96, *SD* = 1.18), and future outlook (*M* = 3.65, *SD* = 0.71). Past-year suicide thoughts were reported by 62.3% (*n* = 198) of TNB youth, and 22.6% (*n* = 72) reported engaging in past-year suicide attempts.

### 3.2. RQ_2_: Latent Class Analysis of Pronoun Affirmation Contexts

Given the variability in pronoun affirmation across contexts, we next examined whether distinct latent classes of pronoun affirmation emerged across social contexts. A latent class analysis (LCA) was conducted using five binary indicators of pronoun affirmation: home, online school, in-person school, friends, and extended family. Models with two through five classes were estimated. As shown in Table 2, the 3-class solution was retained, having a lower BIC than the 2-class solution, acceptable entropy (0.724), strong average posterior probabilities across classes (0.993, 0.970, 0.888), and interpretable class profiles. The 4- and 5-class solutions produced higher BIC values and less interpretable profiles. Class characteristics, affirmation indicator probabilities, and outcome descriptives are presented in Table 3. The latent class profiles are depicted in Figure 1.

The first class, predominantly friends affirmation (27.3%, *n* = 87), was characterized by low probabilities of affirmation in school contexts (online school: 7.4%; in-person school: 6.4%), home (23.8%), and extended family (11.5%). Friend affirmation was high in this class (85.8%). The second class, non-familial affirmation (55.8%, *n* = 178), was characterized by high probabilities of school and peer affirmation (online school: 92.3%; in-person school: 92.3%; friends: 98.5%), low home affirmation (27.4%), and near-zero extended family affirmation (0.6%). The third class, comprehensive affirmation (16.9%, *n* = 54), was characterized by high probabilities of affirmation across all contexts, including home (94.2%), school (online: 100%; in-person: 99.6%), friends (100%), and extended family (70.1%). In other words, among these three classes, predominantly friends affirmation reflected low affirmation across most contexts except friends; non-familial affirmation reflected high school and peer affirmation with limited family affirmation; and comprehensive affirmation reflected high affirmation across all contexts (i.e., school, peers, and family).

### 3.3. RQ_3_: Demographic Differences Across Pronoun Affirmation Classes

Chi-squared analyses examined whether demographic characteristics differed across pronoun affirmation classes (see Table 4). Results indicated a significant association between class membership and transmasculine identity, χ^2^(2) = 8.21 (*p* = 0.017). Transmasculine youth were more represented in comprehensive affirmation (39.2%) compared to the predominantly friends affirmation (21.2%) and non-familial affirmation (20.3%) classes, suggesting that transmasculine youth may have greater access to multiple affirming contexts. However, pairwise comparisons indicated that only the difference between non-familial and comprehensive affirmation was significant after Bonferroni correction (*p* = 0.010); however, the difference between predominantly friends affirmation and comprehensive affirmation was not significant. Results also indicated a significant association between class membership and nonbinary identity, χ^2^(2) = 27.66 (*p* < 0.001). Nonbinary youth were less represented in comprehensive affirmation (51.0%) compared to predominantly friends affirmation (84.7%) and non-familial affirmation (83.6%), suggesting that nonbinary youth may have less access to affirmation across family contexts. No significant differences were observed for transfeminine youth identity, Youth of Color status, plurisexual identity, disability status, or age.

### 3.4. RQ_4_: Associations Between Pronoun Affirmation Classes and Mental Health Outcomes

Linear and logistic regression models examined associations between affirmation class membership and outcomes, controlling for transmasculine identity, transfeminine identity, plurisexual identity, Youth of Color status, age, and disability status. Predominantly friends affirmation class served as the reference group in order to examine whether more diverse affirmation contexts are associated with better mental health outcomes (see Table 5).

**Class Differences.** As shown in Table 3, one-way ANOVAs indicated significant class differences in depressive symptoms (*F*(2, 315) = 12.04, *p* < 0.001), anxiety symptoms (*F*(2, 315) = 7.47, *p* < 0.001), and future outlook (*F*(2, 315) = 7.11, *p* < 0.001). Post hoc Tukey HSD comparisons indicated that youth in non-familial affirmation and comprehensive affirmation reported significantly lower depressive and anxiety symptoms than youth in predominantly friends affirmation. For future outlook, youth in non-familial affirmation reported significantly greater future outlook than youth in predominantly friends affirmation. Non-familial affirmation and comprehensive affirmation did not differ significantly from each other on any continuous outcome. Chi-squared analyses indicated a significant association between class membership and suicide thoughts (χ^2^(2) = 19.42, *p* < 0.001). Rates of suicide thoughts were highest in predominantly friends affirmation (75.9%), followed by non-familial affirmation (62.7%), and lowest in comprehensive affirmation (38.9%). Class membership was not significantly associated with suicide attempts. The outcome descriptives and test statistics are presented in Table 3.

**Depressive and Anxiety Symptoms.** After adjusting for covariates, the overall model for depressive symptoms was significant (*F*(8, 303) = 4.94, *p* < 0.001), explaining 11% of the variance. Compared to predominantly friends affirmation, youth in non-familial affirmation reported significantly lower depressive symptoms (β = −0.25, *B* = −0.58, *SE* = 0.14, *p* < 0.001), as did youth in comprehensive affirmation (β = −0.27, *B* = −0.82, *SE* = 0.20, *p* < 0.001). The overall model for anxiety symptoms was significant (*F*(8, 303) = 4.99, *p* < 0.001), explaining 12% of the variance. Compared to predominantly friends affirmation, youth in non-familial affirmation reported significantly lower anxiety symptoms (β = −0.21, *B* = −0.49, *SE* = 0.15, *p* < 0.001), as did youth in comprehensive affirmation (β = −0.22, *B* = −0.69, *SE* = 0.20, *p* < 0.001). Youth with disabilities reported more depressive symptoms (β = 0.18, *B* = 0.43, *SE* = 0.13, *p* < 0.01) and more anxiety symptoms (β = 0.24, *B* = 0.57, *SE* = 0.14, *p* < 0.001).

**Future Outlook.** The overall model for future outlook was significant (*F*(8, 303) = 2.44, *p* < 0.01), explaining 6% of the variance. Compared to predominantly friends affirmation, youth in non-familial affirmation reported significantly better future outlook (β = 0.23, *B* = 0.32, *SE* = 0.09, *p* < 0.001), as did youth in comprehensive affirmation (β = 0.17, *B* = 0.32, *SE* = 0.13, *p* < 0.05).

**Suicide Thoughts and Attempts.** In logistic regression models, youth in non-familial affirmation had significantly lower odds of suicidal ideation compared to youth in predominantly friends affirmation (AOR = 0.50, 95% CI [0.27, 0.90], *p* < 0.05). Youth in comprehensive affirmation also had significantly lower odds of suicidal ideation compared to youth in predominantly friends affirmation (AOR = 0.19, 95% CI [0.09, 0.41], *p* < 0.001). Class membership was not significantly associated with suicide attempt. Disability status was associated with greater odds of suicide attempt (AOR = 2.65, 95% CI [1.46, 4.92], *p* < 0.01). Plurisexual identity was associated with significantly lower odds of suicide attempt (AOR = 0.48, 95% CI [0.26, 0.89], *p* < 0.05). Youth of Color status was associated with significantly greater odds of suicide attempt (AOR = 2.97, 95% CI [1.63, 5.54], *p* < 0.001).

To test whether comprehensive affirmation was associated with significantly better outcomes than non-familial affirmation, regression models were re-estimated with non-familial affirmation as the reference group. Comprehensive affirmation was associated with significantly lower odds of suicidal ideation compared to non-familial affirmation (AOR = 0.38, 95% CI [0.19, 0.74], *p* < 0.01). However, the two classes did not differ significantly on depressive symptoms, anxiety symptoms, or future outlook (all *p* > 0.15), indicating that the mental health advantages of comprehensive over non-familial affirmation are specific to suicidal ideation rather than being reflective of broader mental health improvements.

### 3.5. Context-Specific Analyses

Additionally, two sets of variable-centered analyses examined associations between pronoun affirmation in each socioecological context and resilience outcomes. Model A estimated separate regression models for each context alongside covariates. Model B entered all five contexts simultaneously (*n* = 213 due to listwise deletion). Results are presented in Table 6.

**Depressive and Anxiety Symptoms.** In Model A, affirmation at home (β = −0.17, *B* = −0.39, *SE* = 0.14, *p* < 0.01), online school (β = −0.32, *B* = −0.77, *SE* = 0.15, *p* < 0.001), in-person school (β = −0.27, *B* = −0.64, *SE* = 0.14, *p* < 0.001), with friends (β = −0.20, *B* = −1.04, *SE* = 0.28, *p* < 0.001) was associated with fewer depressive symptoms. In Model B, only home affirmation remained significant (β = −0.21, *B* = −0.48, *SE* = 0.18, *p* < 0.01).

In Model A, affirmation at home (β = −0.16, *B* = −0.39, *SE* = 0.14, *p* < 0.01), online school (β = −0.22, *B* = −0.55, *SE* = 0.15, *p* < 0.001), in-person school (β = −0.19, *B* = −0.47, *SE* = 0.15, *p* < 0.01), and with friends (β = −0.20, *B* = −1.09, *SE* = 0.29, *p* < 0.001) were each associated with fewer anxiety symptoms. In Model B, none of the contexts were significant.

**Future Outlook.** In Model A, affirmation in online school (β = 0.22, *B* = 0.33, *SE* = 0.09, *p* < 0.001), in-person school (β = 0.19, *B* = 0.29, *SE* = 0.09, *p* < 0.01), and with friends (β = 0.16, *B* = 0.51, *SE* = 0.18, *p* < 0.01) were associated with better future outlook. In Model B, none of the contexts were significant.

**Suicide Thoughts and Attempts.** In Model A, affirmation in online school was associated with lower odds of suicidal thoughts (*AOR* = 0.47, 95% *CI* [0.25, 0.85], *p* < 0.05). Affirmation in in-person school (*AOR* = 0.41, 95% *CI* [0.23, 0.73], *p* < 0.01) and with extended family (*AOR* = 0.37, 95% *CI* [0.19, 0.72], *p* < 0.01) was also associated with lower odds of suicide thoughts. In Model B, only in-person school affirmation remained significantly associated with lower odds of suicide thoughts (*AOR* = 0.30, 95% *CI* [0.09, 0.88], *p* < 0.05). Pronoun affirmation was not significantly associated with suicide attempt across any individual context in Model A or Model B. Given the small event counts per class, suicide attempt models were likely underpowered.

## 4. Discussion

The current study examined how latent patterns of pronoun affirmation across multiple social contexts relate to mental health outcomes (i.e., depressive and anxiety symptoms, future outlook, suicide thoughts and attempts) among TNB youth. Consistent with prior research on gender affirmation via chosen name and pronoun use (Russell et al., 2018; Sequeira et al., 2022), our findings demonstrate that pronoun affirmation is a consistent promotive factor for mental health among TNB youth. Specifically, our study demonstrates that pronoun affirmation classes with more diverse contexts were associated with fewer depressive and anxiety symptoms, a more positive future outlook, and lower odds of suicide thoughts. Our study is the first to examine latent patterns of pronoun affirmation across social contexts, highlighting that a range of affirmation, rather than a single source of affirmation, is particularly important for youth mental health and well-being. Taken together, these findings suggest that social contexts are not static spaces in which TNB youth development occurs, but rather active and dynamic spaces where pronoun affirmation can foster resilience and opportunities for TNB youth to thrive.

Specific to our research questions, pronoun affirmation differed across contexts, with affirmation most common with friends and least common with extended family (*RQ1*). Further, three distinct latent classes of affirmation emerged, characterized by predominantly friend, school-and-friend, and multi-context affirmation patterns (*RQ2*). These classes differed by transmasculine and nonbinary identity but not by other demographic characteristics examined (*RQ3*). Finally, more diverse affirmation classes were associated with better mental health outcomes across nearly all indicators (*RQ4*), and individual contexts of affirmation showed distinct patterns of association with outcomes, with school and extended-family affirmation showing the most extensive associations across outcomes (*RQ5*).

### 4.1. Contexts Matter for Pronoun Affirmation and Resilience

Aligning with minority stress theory (Brooks, 1981; Meyer, 2003; Toomey, 2021), our study found that pronoun affirmation of TNB youth may promote resilience and offer opportunities to reduce disparities related to minority stressors within cisheteronormative systems. Yet, while pronoun affirmation might help to reduce the risk of thinking about suicide, it might not be enough to reduce the risk of youth engaging in suicide attempts (Johns et al., 2019). Importantly, a pattern emerged across affirmation classes, with the comprehensive affirmation class showing the greatest reductions in suicide thoughts, followed by non-familial affirmation, relative to the predominantly friends affirmation class. This finding is consistent with resilience frameworks and minority stress theory, which suggests that promotive factors that accumulate across multiple social contexts may provide compounding resilience benefits for TNB youth (Toomey, 2021). Further, comprehensive affirmation included youth who experienced pronoun affirmation within the contexts of their families, while this context of affirmation was much less likely among youth in predominantly friends affirmation and non-familial affirmation. This finding suggests that family-based pronoun affirmation is a critical leverage point for promoting resilience and opportunities to thrive among TNB youth. Indeed, rates of suicide thoughts among comprehensive affirmation youth (38.9%) were less than half those observed in predominantly friends affirmation (75.9%), a reduction of more than 37%. This finding underscores that multicontextual affirmation, particularly when it includes family, may offer protection against suicide thoughts among TNB youth; however, longitudinal research is needed to further confirm the directionality of these associations.

Examining affirmation profile contexts revealed that youth in the non-familial affirmation class showed significantly better mental health outcomes compared to the predominantly friends affirmation class across all depressive and anxiety symptoms, future outlook, and suicide thoughts. Non-familial affirmation was characterized by high probabilities of school and peer affirmation but low home and extended family affirmation, which underscores the particular importance of institutional affirmation during adolescence. These findings are consistent with research linking school belonging and inclusive school environments to better mental health outcomes among TNB youth (GLSEN, 2023; Russell & Fish, 2016) and carry profound importance within the current sociopolitical climate. Moreover, online and in-person school affirmation showed nearly identical associations with outcomes, suggesting that the modality of school affirmation may matter less than whether affirmation occurs in school contexts at all. Recent legislative efforts to restrict pronoun affirmation within schools (GLSEN, 2023; Movement Advancement Project, 2025) have potentially compounding effects on TNB youth mental health, spanning internalized distress, future outlook, and suicide risk.

Critically, the direct comparison of comprehensive and non-familial affirmation classes revealed that family affirmation was associated with a specific rather than broad additional association with mental health outcomes. Comprehensive affirmation youth had significantly lower odds of suicide thoughts relative to non-familial affirmation youth (*AOR* = 0.38), but the two classes did not differ significantly on depressive symptoms, anxiety symptoms, or future outlook. This suggests that school and peer affirmation may be associated with comparable general mental health among TNB youth, but that family affirmation may be uniquely associated with suicide risk specifically. Given that family affirmation was near zero for non-familial affirmation youth (0.6%), these findings point to the family as the most critical and underutilized context for intervention.

### 4.2. Intersectionality, Affirmation, and Resilience

Consistent with our use of intersectionality, the findings presented here offer insight into how identity might shape access to affirmation across social contexts, while acknowledging that this approach cannot capture the full complexity of intersecting identities. Class differences in affirmation revealed that transmasculine youth were more likely to be represented in comprehensive affirmation, the highest affirmation class, suggesting transmasculine youth may have greater access to affirmation across family, peer, and school contexts. While prior research has documented variability in affirmation experiences across TNB subgroups (The Trevor Project, 2022, 2024), the specific factors driving differential access to family affirmation by gender identity subgroup remain understudied. Future research is needed to examine whether this finding reflects broader sociocultural patterns in which binary transmasculine identities are more readily affirmed by family members, or other contextual factors (e.g., age of coming out).

Results also indicated that nonbinary youth were less likely to be represented in comprehensive affirmation (51.0%) compared to predominantly friends affirmation (84.7%) and non-familial affirmation (83.6%), χ^2^(2) = 27.66, *p* < 0.001, suggesting that nonbinary youth may have less access to affirmation across family contexts. This finding is consistent with research documenting higher rates of invalidation and poorer mental health outcomes among nonbinary individuals (Johnson et al., 2024). It may also reflect the additional challenges nonbinary youth face in contexts where youth face expectations to uphold binary gender norms.

Our findings, interpreted through an intersectional lens, highlight the unique nature of mental health disparities and opportunities for resilience experienced by TNB youth with disabilities, Youth of Color, and TNB youth with minoritized gender and sexual identities. Across models, TNB youth with disabilities reported higher depressive and anxiety symptoms, as well as greater odds of suicide thoughts and attempts. Disability status was a significant predictor across several class-based and context-specific models, indicating that the mental health disparities experienced by TNB youth with disabilities persist even when accounting for pronoun affirmation. This finding suggests the need for more research documenting the intersections of disability and TNB identities, particularly as they relate to unique supports and accommodations.

Similarly, TNB Youth of Color had nearly three times greater odds of suicide attempts compared to White TNB youth. Consistent with prior research (Bauer et al., 2021), this finding brings additional attention to the layered implications of racism and transphobia for mental health outcomes among TNB youth and emphasizes the critical need to consider intersecting identities and experiences (Crenshaw, 1991; Santos & Toomey, 2018). Plurisexual identity was associated with lower odds of suicide attempts, a finding that should be interpreted cautiously and explored in future research. Together, these findings highlight the necessity of implementing nuanced, intersectional approaches that recognize the complex experiences of marginalization faced by TNB youth with disabilities and TNB Youth of Color.

### 4.3. Implications for Interventions

This study contributes to research demonstrating that pronoun affirmation contributes to mental health outcomes for TNB youth. Our use of LCA provides new insight into how distinct patterns of pronoun affirmation across contexts may be particularly promotive for TNB youths’ positive outcomes. Implications for intervention programs include potential for increasing family and peer involvement for greater pronoun affirmation across multiple social contexts. Family- and school-based programs offer opportunities to train educators, parents, and peers on the benefits of affirmative pronoun use and to practice using nonbinary pronouns (e.g., they/them) when they are not with the TNB youth. The policy implications of our research are timely given current legislative efforts to restrict gender-affirming practices in schools. Results suggest that policies restricting pronoun affirmation may reduce access to affirming contexts that are associated with resilience among TNB youth. Conversely, policies that protect and promote pronoun affirmation across contexts (e.g., allowing school staff to use students’ chosen pronouns) may serve as important upstream efforts to protect TNB youth from negative outcomes.

Our findings highlight that pronoun affirmation is particularly low in family settings. This suggests that family therapists and practitioners should be encouraged to consider and adopt strategies to help families affirm their TNB family members’ pronouns. Family-based interventions should include opportunities for parents to practice affirming their TNB child’s pronouns (e.g., role-play with the therapist) and to navigate mistakes (e.g., unintentional misgendering). School-based interventions should also include supportive initiatives to foster pronoun affirmation and inclusion (e.g., training school staff on how to affirm students). Given the findings of the current study and extant research, these efforts will likely show that pronoun affirmation is associated with better mental health.

### 4.4. Limitations and Future Directions

This study is not without limitations. First, the findings are based on cross-sectional data; thus, we cannot conclude causality between pronoun affirmation and mental health. Relatedly, effect sizes were small to moderate (e.g., the model predicting future outlook accounted for only 6% of the variance). These associations should be interpreted as having modest practical significance. Future longitudinal studies are needed to address concerns about directionality in this association. In particular, daily diary study designs may be particularly helpful for capturing fluctuations in mental health outcomes on days or in moments when individuals affirm (or reject) a TNB youth’s pronouns. Second, the measure that assessed pronoun affirmation context did not measure the quality of affirmation received (e.g., passive acceptance) or the salience of the context (e.g., time spent in given context). Future research is needed to understand how the coalescence of quality and salience of affirmation shapes the extent to which pronoun affirmation is most impactful for TNB youth outcomes, and to determine which contexts have the greatest potential for informing targeted interventions. Third, suicide thoughts and suicide attempts were each assessed with a single item, and although single-item suicide measures are commonly used in population-based adolescent research and have demonstrated validity in prior studies with sexual and gender minority youth (Johns et al., 2019; Price & Green, 2023), future research would benefit from using multi-item assessments of suicide risk that capture severity and frequency. The suicide attempt models were further limited by small cell sizes, notably in the comprehensive affirmation class (*n* = 12), which constrained statistical power. Fourth, although our sensitivity analysis confirmed that including the work context did not substantially change the classes that emerged, findings may not fully capture affirmation experiences among employed TNB youth. Fifth, pronoun affirmation among friends showed a ceiling effect (94.9% affirmed), which may have attenuated associations in the variable-centered models. Future research should examine more nuanced dimensions of peer affirmation (e.g., type of relationships, frequency of affirmation, type of affirmation) to capture meaningful variation.

Finally, although the sample size was sufficient for analyses, certain subgroups were underrepresented in the sample. Transfeminine youth were a small proportion of the sample, which limited statistical power in context-specific regression models for suicide attempts among this subgroup; these findings should be interpreted with caution. Similarly, comprehensive affirmation included only 54 participants, and findings specific to this class, particularly for suicide outcomes, should be replicated in larger, more diverse samples.

Additionally, while nonbinary youth were significantly less represented in comprehensive affirmation (51.0%) compared to predominantly friends affirmation (84.7%) and non-familial affirmation (83.6%), the factors driving this pattern remain unclear. Future research should examine whether this reflects differential access to family affirmation specifically, structural barriers to multicontextual affirmation for nonbinary youth, or other contextual factors such as cultural contexts. Future research is needed to consider intervention program efforts, including school- and family-based prevention programs aimed at promoting resilience among TNB youth. Although national data indicate that 95% of U.S. adolescents have smartphone access with minimal variation by household income (Faverio & Sidoti, 2024), recruitment via Qualtrics Panel Services constitutes a non-probability sample with monetary compensation. TNB youth who are unable to participate in online surveys may remain underrepresented, and future research should employ community-based or school-based recruitment strategies. Additionally, although intersectionality informed our interpretations, our methodological approach entered social identities as separate covariates and class-distribution comparisons rather than modeling intersecting social positions directly (e.g., via interaction terms). This is a common limitation of quantitative intersectionality work (Bauer et al., 2021) and should be addressed in future research with larger samples that can support this approach. Further, several demographic subgroups in this study were small, and non-significant class differences (e.g., race/ethnicity, sexual orientation, and disability status) should not be interpreted as evidence that no disparities exist. Rather, this study was underpowered to detect differences. Future research is also needed to more comprehensively examine intersecting identities among TNB youth, particularly among TNB disabled youth and Youth of Color.

## 5. Conclusions

This study demonstrates that distinct patterns of chosen pronoun affirmation across multiple social contexts are associated with positive outcomes among TNB youth, including lower depressive and anxiety symptoms, greater future outlook, and fewer suicide thoughts. These findings suggest that programs, interventions, and policies that support pronoun affirmation may be important for addressing structural inequities experienced by TNB youth. Our findings underscore the need for intersectional approaches to addressing inequities in resilience among TNB youth, particularly by considering how affirmation within multiple contexts, including schools, peer relationships, and families, may buffer against the negative impacts of structural stressors. Importantly, while school and peer affirmation showed consistent associations with mental health outcomes, family affirmation appeared to carry unique promotive significance for suicide risk reduction specifically, which has direct implications for family-based intervention and practice. As legislation continues to restrict gender-affirming practices, including pronoun affirmation within schools, our findings underscore the importance of policies and practices that support pronoun affirmation across the contexts where TNB youth learn, develop, and navigate adolescence.

## Figures and Tables

**Figure 1:**
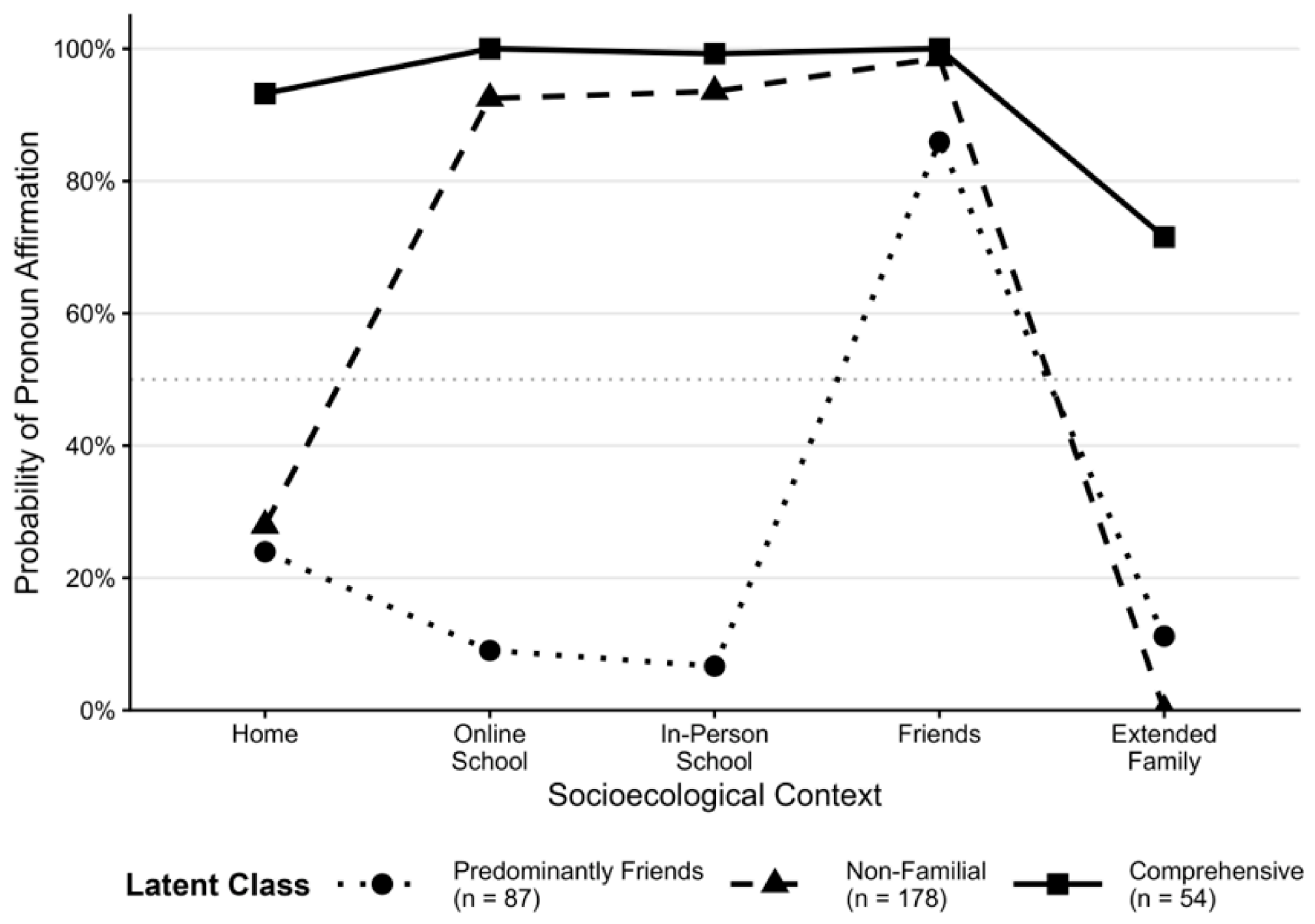
Latent Classes of Pronoun Affirmation Across Socioecological Contexts. *Note.* Probabilities reflect model-estimated item-response probabilities from the latent class analysis. Predominantly friends affirmation was characterized by high affirmation among friends and low affirmation across all other contexts. Non-familial affirmation was characterized by high school and peer affirmation with near-zero extended family affirmation. Comprehensive affirmation was characterized by high affirmation across all five contexts, including home and extended family.

**Table 1:** Descriptive Statistics of Pronoun Affirmation Across Contexts.

	Applicable (*n*)	Affirmed (*n*)	Affirmed (%)
At home	310	128	41.3%
Online school	245	163	66.5%
In-person school	265	177	66.8%
With friends	313	297	94.9%
Extended family	296	57	19.3%

*Note.* Applicable *n* reflects the number of participants for whom each context was relevant. Affirmed % reflects the proportion of applicable participants who reported pronoun affirmation in that context.

**Table 2:** Model Fit Statistics of Estimated Latent Classes.

Model	Solution	Log Likelihood	BIC	AIC	Entropy	Smallest Group %
1	2-class	−651.17	1365.76	1324.34	0.758	33.9%
**2**	**3-class**	**−626.71**	**1351.42**	**1287.41**	**0.724**	**16.9%**
3	4-class	−612.56	1357.80	1271.12	0.719	9.1%
4	5-class	−611.91	1391.00	1281.81	0.709	2.5%

*Note*. BIC = Bayesian information criterion; AIC = Akaike’s information criterion. Entropy reflects classification certainty (range 0–1; higher = better). Number of parameters: 2-class (11), 3-class (17), 4-class (23), 5-class (29). Bolding indicates the retained solution, selected based on lower BIC relative to the 2-class solution, acceptable entropy (0.724), strong average posterior probabilities (0.993, 0.970, 0.888), and interpretable profiles. The 4- and 5-class solutions produced higher BIC values and less interpretable classes.

**Table 3:** Latent Class Characteristics and Mental Health Outcome Descriptives.

	Predominantly Friends Affirmation		Non-Familial Affirmation		Comprehensive Affirmation		Test Statistic	
	(*n* = 87)		(*n* = 178)		(*n* = 54)			
Affirmation Indicators:	%	*M (SD)*	%	*M (SD)*	%	*M (SD)*		
At home	23.8% _a_		27.4% _a_		94.2% _b_		χ^2^(2) = 81.21 ***	
Online school	7.4% _a_		92.3% _b_		100.0% _b_		χ^2^(2) = 195.32 ***	
In-person school	6.4% _a_		92.3% _b_		99.6% _b_		χ^2^(2) = 210.96 ***	
With friends	85.8% _a_		98.5% _b_		100.0% _b_		χ^2^(2) = 31.71 ***	
Extended family	11.5% _a_		0.6% _b_		70.1% _c_		χ^2^(2) = 234.63 ***	
**Mental Health Outcomes:**								
Depressive symptoms		3.61 _a_ (1.01)		3.00 _b_ (1.11)		2.79 _b_ (1.20)		F(2, 315) = 12.04 ***, η^2^ = 0.071
Anxiety symptoms		3.36 _a_ (1.13)		2.84 _b_ (1.14)		2.70 _b_ (1.25)		F(2, 315) = 7.47 ***, η^2^ = 0.045
Future outlook		3.41 _a_ (0.75)		3.76 _b_ (0.66)		3.69 _a,b_ (0.73)		F(2, 315) = 7.11 ***, η^2^ = 0.043
Suicide thoughts	75.9% _a_ (66)		62.7% _a_ (111)		38.9% _b_ (21)		χ^2^(2) = 19.42 ***, V = 0.234	
Suicide attempts	29.9% _a_ (26)		19.2% _a_ (34)		22.2% _a_ (12)		χ^2^(2) = 3.80, V = 0.075	

*Note.* Affirmation indicators reflect item-response probabilities for each class. *M (SD)*— means and standard deviations are presented for continuous outcomes; percentages and frequencies are presented for dichotomous outcomes. *F-test* is provided for continuous outcomes. Chi-squared (χ^2^) test is provided for dichotomous outcomes. η^2^ = eta-squared; V = Cramér’s V. Subscripts of the same letter within a row indicate no statistically significant difference based on comparisons. Bolding indicates significance. *** *p* < 0.001.

**Table 4:** Demographic Differences Across Latent Classes.

	Predominantly Friends Affirmation	Non-Familial Affirmation	Comprehensive Affirmation	Test Statistic
Transmasculine	21.2% _a_	20.3% _a_	39.2% _b_	χ^2^(2) = 8.21 (2) *, V = 0.141
Transfeminine	7.1% _a_	9.6% _a_	17.6% _a_	χ^2^(2) = 4.06 (2), V = 0.081
Nonbinary	84.7% _a_	83.6% _a_	51.0% _b_	χ^2^(2) = 27.66 (2) **, V = 0.287
Youth of Color	37.9% _a_	42.7% _a_	50.0% _a_	χ^2^(2) = 1.99 (2), V = 0.000
Plurisexual identity	72.4% _a_	75.8% _a_	66.7% _a_	χ^2^(2) = 1.84 (2), V = 0.000
Disability status	41.4% _a_	41.0% _a_	33.3% _a_	χ^2^(2) = 1.14 (2), V = 0.000
Age (*M*, *SD*)	18.6 (2.28)	18.6 (2.21)	18.9 (1.90)	*F*(2, 316) = 0.51

*Note.* Percentages reflect proportions of participants in each class identifying with each characteristic. *M* = mean; *SD* = standard deviation; χ^2^ = Chi-squared; V = Cramér’s V. Plurisexual (0 = no, 1 = yes), Youth of Color (0 = not Youth of Color, 1 = Youth of Color), Age, Disability (0 = not disabled, 1 = disabled). Subscripts of the same letter within a row indicate no statistically significant difference between classes. Age was continuous and tested with one-way ANOVA. Bolding indicates significance. * p < 0.05, ** *p* < 0.001.

**Table 5:** Regression Models Predicting Mental Health Outcomes by Pronoun Affirmation Class.

	Linear Regressions			Logistic Regressions	
	Depressive Symptoms *B* (*SE*)	Anxiety Symptoms *B* (*SE*)	Future Outlook *B* (*SE*)	Suicide Thoughts *AOR* [95% *CI*]	Suicide Attempts *AOR* [95% *CI*]
Non-Familial Affirmation *^a^*	−0.58 (0.14) ***	−0.49 (0.15) ***	0.32 (0.09) ***	0.50 [0.27, 0.90] *	0.53 [0.28, 1.00]
Comprehensive Affirmation *^a^*	−0.82 (0.20) ***	−0.69 (0.20) ***	0.32 (0.13) *	0.19 [0.08, 0.41] ***	0.59 [0.24, 1.37]
Transmasculine	0.06 (0.15)	0.28 (0.16) ^†^	−0.18 (0.10) ^†^	1.01 [0.57, 1.84]	1.12 [0.56, 2.16]
Transfeminine	0.13 (0.21)	0.16 (0.21)	−0.12 (0.13)	1.21 [0.54, 2.80]	1.48 [0.60, 3.44]
Youth of Color	0.06 (0.13)	0.06 (0.13)	−0.00 (0.08)	1.45 [0.87, 2.43]	2.97 [1.63, 5.54] ***
Plurisexual identity	−0.26 (0.14) ^†^	−0.17 (0.15)	0.09 (0.09)	0.93 [0.53, 1.61]	0.48 [0.26, 0.89] *
Age	−0.03 (0.03)	−0.02 (0.03)	0.02 (0.02)	0.94 [0.84, 1.05]	1.02 [0.90, 1.17]
Disability status	0.43 (0.13) ***	0.57 (0.14) ***	−0.04 (0.08)	2.29 [1.36, 3.93] **	2.65 [1.46, 4.92] **
*R*^2^/*NR*^2^Δ	0.115	0.117	0.061	0.044	0.011
*Cohen’s f* ^2^	0.074	*f*^2^ = 0.049	0.043	-	-

*Note. ^a^* Compared to reference group = predominantly friends affirmation. *B* = unstandardized coefficient, *SE* = standard error, *AOR* = adjusted odds ratio, 95% CI = confidence interval. *R*^2^ = multiple R-squared; *NR*^2^Δ = Nagelkerke *R*^2^ change for class beyond covariates; *Cohen’s f*^2^ = incremental effect size. Plurisexual (0 = no, 1 = yes), Youth of Color (0 = not Youth of Color, 1 = Youth of Color), Age (continuous), Disability (0 = not disabled, 1 = disabled). Nonbinary identity was the referent group for gender identity. Monosexual identity (i.e., gay, lesbian) was the referent group for sexual identity. White was the referent group for race/ethnicity. Bolding indicates significance. ^†^ *p* < 0.10, * *p* < 0.05, ** *p* < 0.01, *** *p* < 0.001.

**Table 6:** Affirmation Context Models Predicting Mental Health Outcomes.

	Linear Regressions			Logistic Regressions	
	Depressive Symptoms *B (SE)*	Anxiety Symptoms *B (SE)*	Future Outlook *B (SE)*	Suicide Thoughts *AOR [95% CI]*	Suicide Attempts *AOR [95% CI]*
**Model A: Individual Context Models**					
At Home	−0.39 (0.14) ***	−0.39 (0.14) **	0.08 (0.11)	0.71 [0.43, 1.17]	1.12 [0.62, 2.03]
Online School	−0.77 (0.15) ***	−0.55 (0.15) ***	0.33 (0.09) ***	0.47 [0.25, 0.85] *	0.56 [0.28, 1.14]
In-person School	−0.64 (0.14) ***	−0.47 (0.15) **	0.29 (0.09) **	0.41 [0.23, 0.73] **	0.56 [0.30, 1.07] ^†^
With Friends	−1.04 (0.28) ***	−1.09 (0.29) ***	0.51 (0.18) **	0.50 [0.13, 1.52]	0.49 [0.16, 1.60]
Extended Family	−0.35 (0.18) ^†^	−0.34 (0.18) ^†^	0.11 (0.11)	0.37 [0.19, 0.72] **	0.87 [0.39, 1.87]
**Model B: All Contexts Simultaneously**					
At Home	**−** 0.48 (0.18) **	−0.33 (0.19) ^†^	0.04 (0.12)	0.78 [0.37, 1.68]	1.03 [0.41, 2.462]
Online School	−0.30 (0.25)	−0.08 (0.26)	0.23 (0.17)	1.48 [0.51, 4.76]	0.98 [0.27, 3.65]
In-person School	−0.39 (0.25)	−0.38 (0.26)	0.06 (0.17)	0.30 [0.09, 0.88] *	0.55 [0.15, 1.93]
With Friends	−0.60 (0.35) ^†^	−0.72 (0.37) ^†^	0.17 (0.24)	0.85 [0.16, 3.73]	0.92 [0.19, 5.65]
Extended Family	0.12 (0.22)	−0.04 (0.23)	0.09 (0.15)	0.62 [0.25, 1.56]	0.94 [0.31, 2.75]

*Note.* Model A presents separate regression models for each context entered alongside covariates; each row is an independent model. Model B enters all five contexts simultaneously alongside covariates (*n* = 213 due to listwise deletion). *B* = unstandardized coefficient; *SE* = standard error; *AOR* = adjusted odds ratio; 95% CI = confidence interval. Bolding indicates significance. ^†^ *p* < 0.10, * *p* < 0.05, ** *p* < 0.01, *** *p* < 0.001.

## Data Availability

The data are not publicly available but may be available from the corresponding author upon reasonable request to the study PI, Russell B. Toomey (toomey@arizona.edu) and subject to institutional approval.

## Supplementary Materials

The following supporting information can be downloaded at: https://www.mdpi.com/article/10.3390/bs16101756/s1, Table S1: Model Fit Statistics of Latent Class Analysis Including Work Context. Table S2: Latent Classes Including Work Context.
