# Supplementary material for: Patterns of Pronoun Affirmation Across Social Contexts and Opportunities for Resilience Among Trans and Nonbinary Youth
Source: Behav Sci (Basel). 2026 Sep 27;16(10):1756. doi: 10.3390/bs16101756 (PMC13701966; doi:10.3390/bs16101756)
Supplement: Supplementary file 1 [file behavsci-16-01756-s001.zip › behavsci-4440966-supplementary.pdf]

Supplementary materials

S1. Table S1

Supplemental Table S1. Model Fit Statistics of Latent Class Analysis Including Work Context

Model	Solution	Log likelihood	Number of parameters	BIC	AIC	Entropy	Smallest group %
1	2-class	-764.54	13	1604.018	1555.070	0.785	34.2%
2	**3-class**	-725.58	20	1566.470	1491.167	0.765	25.2%
3	4-class	-711.12	27	1577.906	1476.245	0.815	2.8%
4	5-class	-706.40	34	1608.735	1480.718	0.850	1.2%

Note. BIC = Bayesian information criterion; AIC = Akaike's information criterion. Entropy reflects classification certainty (range 0-1; higher = better). Bolding indicates the retained 3-class solution, based on lower BIC relative to the two-class solution, acceptable entropy, and interpretable profiles. The four- and five-class solutions produced higher BIC values and very small minimum class sizes.

S1. Table S2

Supplemental Table S2. Latent Classes Including Work Context

	**Class 1 (*29.5%*)**	**Class 2 (*25.2%*)**	**Class 3 (*44.4%*)**
**Corresponding Class:**	**Predominantly Friends Affirmation**	**Comprehensive Affirmmation**	**Non-familial Affirmation**
At home	18.2%	92.7%	28.0%
Online school	11.3%	89.8%	93.1%
In person school	4.3%	90.8%	95.9%
With friends	85.3%	98.7%	99.1%
Extended family	5.9%	71.6%	0.0%
At Work	5.9%	71.6%	0.0%

Note. The three-class solution including the work context produced profiles similar to the primary analysis. Class 1 corresponded to the predominantly friends affirmation class, characterized by high friend affirmation and low affirmation in all other contexts; Class 2 corresponded to the comprehensive affirmation class, now including high work affirmation alongside high school, peer, and family affirmation; Class 3 corresponded to the non-familial affirmation class, characterized by high school and peer affirmation with near-zero family and work affirmation. The similarity of these profiles to the primary solution (without work context) supports the decision to exclude the work context from the main analyses.
